# Improving creativity performance by short-term meditation

**DOI:** 10.1186/1744-9081-10-9

**Published:** 2014-03-19

**Authors:** Xiaoqian Ding, Yi-Yuan Tang, Rongxiang Tang, Michael I Posner

**Affiliations:** 1 Institute of Neuroinformatics and Laboratory for Body and Mind, Dalian University of Technology, Dalian, China; 2 Department of Psychology, Texas Tech University, Lubbock, TX 79409, USA; 3 Department of Psychology, University of Oregon, Eugene, OR 97403, USA; 4 Department of Psychology, The University of Texas at Austin, Austin, TX 78705, USA

**Keywords:** Creativity, Emotion, Positive affect, Negative affect, Short-term meditation, Integrative body-mind training, Cross-lagged analysis

## Abstract

**Background:**

One form of meditation intervention, the integrative body-mind training (IBMT) has been shown to improve attention, reduce stress and change self-reports of mood. In this paper we examine whether short-term IBMT can improve performance related to creativity and determine the role that mood may play in such improvement.

**Methods:**

Forty Chinese undergraduates were randomly assigned to short-term IBMT group or a relaxation training (RT) control group. Mood and creativity performance were assessed by the Positive and Negative Affect Schedule (PANAS) and Torrance Tests of Creative Thinking (TTCT) questionnaire respectively.

**Results:**

As predicted, the results indicated that short-term (30 min per day for 7 days) IBMT improved creativity performance on the divergent thinking task, and yielded better emotional regulation than RT. In addition, cross-lagged analysis indicated that both positive and negative affect may influence creativity in IBMT group (not RT group).

**Conclusions:**

Our results suggested that emotion-related creativity-promoting mechanism may be attributed to short-term meditation.

## Background

Creativity is a phenomenon whereby something novel (i.e., original and unexpected) and appropriate (i.e., valuable and adaptive concerning task constraints) is created [1], such as an idea, an artistic or literary work, a painting or musical composition, a solution, and an invention. Creativity is essential to the development and advancement of human civilization and plays a crucial role in our cultural life [2]. Hence, researchers among various disciplines have burgeoning interest in the potential for fostering creativity through education and training.

Traditionally, creativity is viewed as a relatively stable individual difference, with some people being regarded as consistently more creative than others [3]. More recently, creativity has been studied as a less stable phenomenon that varies as a function of brief states of the person and situation [2,4]. It is possible to measure creativity in a fast way that allows assessment of states induced by training. As one of the most widely used test of creativity, Torrance Tests of Creative Thinking (TTCT) is easy to administrate in short time [5,6]. It has fewer limitations and cautions to apply, and is more researched and analyzed than any other creativity instrument [6,7]. Many studies examined the predictive validity of the TTCT including elementary education majors, seventh-grade students, and economically disadvantaged elementary school Black children, which increased the TTCT’s credibility as a predictor of creative productivity [8]. A series of follow-up studies at the range from 7- to 22-year interval found that three of the TTCT subscales (fluency, flexibility and originality) correlated significantly (at the .01 level) with creative achievements [9-11]. Moreover, many reanalysis studies of Torrance’s data concluded that the Creative Indexes (fluency, flexibility, and originality) are the best predictors for adult creative achievement [12,13].

The popularity of meditation in the West has led research into its influence on creativity [14]. Previous studies showed that long-term (over years) meditation training enhances creativity as assessed by TTCT [15,16]. For example, open-monitoring meditation, in which an individual is open to perceive and observe any sensation, promotes divergent thinking, a style of thinking that allows many new ideas to be generated [16]. Some recent research showed that short-term meditation has fostering effects on creative thinking. For example, maintaining a mindful and alert state during meditation results in more insight [17]. In addition, Zen practitioners who meditated in the laboratory performed better on the creative test than Zen practitioners who did not meditate [18]. Our pilot work also suggested that around 3 hours IBMT (30 min/session for 7 sessions) can enhance the creative ability as assessed by TTCT [19]. Although studies have shown the positive effect of meditation on creativity, the cognitive mechanisms are largely unknown [20].

Mood represents a transient state that has attracted a great deal of attention as a potential facilitator of creativity [21-23]. Positive affect (PA) produces more fluent and original responses [21,22,24], while negative affect (NA) has the opposite effect [25,26]. For example, PA leads to greater cognitive flexibility and facilitates creative problem solving across a broad range of settings [27]. PA increases creative performance and implementation efficiency, while NA has no effect [28]. In addition, a study with 256 undergraduates shows that PA tends to be induced to create and use categories more inclusively than NA [26]. Results are interpreted in terms of an influence of affect on cognitive organization. PA biases cognitive control mechanisms in ways that facilitate creativity [29] and borderline effects of NA on categorization might result from normal people’s attempts to cope with NA [26]. Hence, previous studies lead some researchers to conclude that PA promotes fluent and original thinking [21,22,24,30], while NA has the opposite effect [25,26].

Meditation is associated with greater emotion regulation [31-33]. For example, 5 days of IBMT has been shown to improve mood and cognitive processes [34]. Four days of mindfulness meditation is effective at reducing anxiety scores and other cognitive manipulations [35]. A similar training regimen improves mood when compared to a sham meditation and control group [28]. Further, four meditation sessions resulted in greater improvements in mood than participants in a sham condition [36]. Other studies showed improvements in mood after brief and single instruction in mindfulness [31,32].

In summary, meditation is associated with enhancing the ability to self-regulate emotions, which has been found to be a key component in cognition [37,38], including creativity [21-23]. In the integrative reviews of creativity studies, it is proposed that PA fosters creativity fluency and originality because of enhanced cognitive flexibility, which may make more diverse connections among ideas, as well as perceive more differences among the items or content, with NA having the opposite effect [22,25]. Therefore, it is possible that short-term meditation increases capacity to self-regulate emotions and thus improves creative performance.

The present study focuses on the cognitive mechanism of meditation on creativity. Short-term IBMT, adopted from traditional Chinese medicine and incorporating the key components of meditation training, was used as a meditation intervention. Instead of using effort to control thoughts, IBMT is designed to facilitate the achievement of a meditative state with a balance and optimization between mind and body [34,39], and further maintain this state to regulate emotion [40]. On the other hand, relaxation training (RT) involves relaxing different muscle groups from the head to abdomen and forces one to concentrate on the feelings of warmth and heaviness [41]. This progressive muscle training helps a participant achieve physical (body) and mental (mind) relaxation and calmness [34,41-43]. Since both RT and IBMT emphasize achieving their desired states through regulating the body and the mind, RT matches IBMT in the training, and thus we chose RT as an active control condition. We used the Positive and Negative Affect Schedule (PANAS) [44] to measures PA and NA, and the TTCT [10] to assess performance of creativity. PANAS and TTCT were measured before and after training to compare the training effects between IBMT and RT. In addition, the reciprocal cross-lagged effects of PANAS (PA and NA) and TTCT were examined before and after IBMT. Cross-lagged analysis is widely recommended for addressing the issue of temporal precedence [45-48]. It helps to test whether the IBMT-regulated emotion has a causal effect on creativity and to rule out alternative causal hypotheses.

Taken together, we hypothesize that compared to RT (i) IBMT will produce greater creativity (indexed by TTCT) (ii) IBMT will improve emotion (indexed by PANAS scales) and (iii) this improved emotion may mediate the change in creativity.

## Materials and methods

### Participants

Forty healthy undergraduates at Dalian University of Technology (DUT) without any meditation or relaxation experiences were recruited. They were evenly and randomly assigned to IBMT group or RT group (20:20). Nineteen participants in the IBMT group (11 males, aged 21 ± 1.6 years old) completed the whole training of 30 min/day for 7 days (3.5 hours in total). The 20 participants in the RT group (10 males, aged 21 ± 1.3 years old) were given the same amount and length of RT [41]. The study was approved by DUT Institutional Review Board and informed consent was obtained from each participant. The consent form explained that participants would complete the PANAS [44] to measure mood state, and the TTCT [10] to assess performance of creativity.

### PANAS

PANAS [44] is a 20-item measure of PA (10 items) and NA (10 items). All items are rated on a Likert-type scale ranging from 1 (very slightly or not at all) to 5 (extremely). Although PANAS can be administered in terms of varying time frames, subjects responded to PANAS items on basis of to what extent they feel a certain way over the past week. The PA scales reflect the extent to which a person feels enthusiastic, active, or alert. The measure has been validated in Chinese [49]; these authors reported that Cronbach’s alphas for the PA and NA subscales were .85 and .83, respectively.

### Creativity assessment

The creativity performance was assessed through TTCT [10], which has been translated into Chinese language and standardized for the usage in China [50]. TTCT has two versions: TTCT-Verbal and TTCT-Figural [51,52]. The creative scalogram in this study consists of two activities (Product Improvement, and Unusual Uses) from TTCT-Verbal and two activities (Picture Completion, and Repeated Figures of Lines) from TTCT-Figural. All participants answered the same questions. Ten minutes were required to complete each activity to generate as many answers as possible.

The four subscales, with descriptions about scoring and the content measured, are listed as following: (a) Fluency, which is the number of relevant responses to the questions, shows the ability to produce and consider many alternatives; (b) Flexibility, which is the (total) number of categories that answers are assigned based on a criteria table or an almost equivalent judgment, shows the ability to produce responses from a wide perspective; (c) Originality, which is the number of statistically infrequent ideas, shows the ability to produce ideas that differ from others’. The scoring procedure counts the most common responses as 0 and all other legitimate responses as 1. The originality lists are prepared for each item on basis of normative data, which are readily memorized by scorers. (d) Elaboration shows the ability to produce ideas in detail [51,52]. For the purpose of this article, Elaboration will not be discussed.

The raw score (fluency, flexibility, originality) in each activity is converted to T-scores according to a formula in TTCT manual [50]. Each total scale score is the sum of its T-scores in the corresponding scale of the four activities. A TTCT score is the sum of three subscales including fluency, flexibility and originality. Each subscale was rated by a single proficient scorer who was blind to the conditions of the participants.

### Procedures

The experimental sessions included pre-training session, training session, and post-training session.

(i) Pre-training session. Before training, the PANAS and TTCT were administered in a group format. First, all the participants completed the PANAS. Second, To avoid the two tasks interference, after PANAS, the participants were given around 30-min break including 15-min rest and then 15-min explanation of the following TTCT tasks. Third, the TTCT was administered. During this session, the administration was performed blind by one psychology Ph.D. who mastered the TTCT [50]-[52] and PANAS. Each participant completed the tests in a partition type desk.

(ii) Training session. The training sessions were intended to help each participant to increase the meditation or relaxation experience. Both IBMT and RT group completed the 7 consecutive days of training with 30 min/per day respectively, total is 3.5 hours. The first training day occurred on a different day after finishing the pre-training session. Firstly, a qualified coach provided participants a free question-and-answer meeting about techniques (IBMT or RT). Secondly, after ensuring the clear grasp of techniques for the novices, the coach guided participants to practice instructions on a compact disc in a harmonious and relaxed atmosphere. The practice was 30 minutes. The IBMT group concentrated on achieving a balanced state of body and mind. The RT group concentrated on the relaxing of different muscle groups and the corresponding feelings of warmth and heaviness. During the practice, the coach observed facial and body cues and gave proper feedback immediately to those who were struggling with the method. Thirdly, thirty minutes later, each participant filled out a questionnaire and evaluated the practice. The coach gave short responses to subjects as required [34,42].

(iii) Post-training session. This session occurred on the next day after the final training day. The procedures of this session were consistent with the pre-session. Participants were given the PANAS firstly, and the TTCT 30 min later.

### Statistical analysis

ANOVA, t tests, linear regression and a cross-lagged panel design were applied for analysis. All analyses were performed using SPSS software.

To examine the homogeneity in TTCT or PANAS between IBMT group and RT group before training, an independent t-test was used to compare the differences between two groups in mean values (TTCT or PANAS). And then, we conducted preliminary analyses using a repeated-measures analysis of variance (ANOVAs) method between groups on each dependent variable (TTCT or PANAS) with time as a factor. When statistically significant effects were found, the independent t-test was used to compare differences in mean values of percent changes from pre to post between two groups.

**Figure 1:**
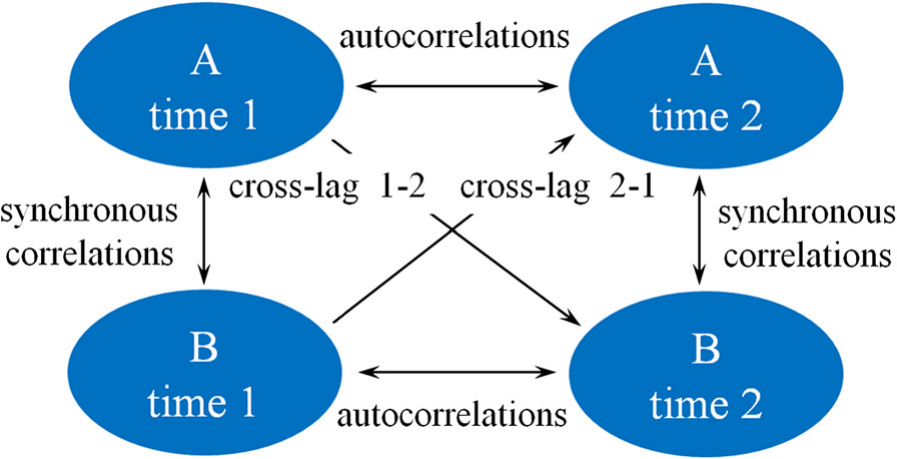
The cross-lagged panel design used to evaluate causal relationships between A and B.

Causal relationships could be inferred by utilizing a cross-lagged panel design, in which variables are collected at least twice [53,54]. The basic results of cross-lagged analyses include a complete correlation matrix: stationarity of correlations (Csta, autocorrelations), synchronous correlations (Csyn), and cross-lagged correlations (Ccl) (Figure 1). First, to assume that a causal model based on crossed-lagged panel correlations is valid, the Csyn and Csta coefficients must be high in magnitude and statistically significant in the non-cross direction [47]. Second, for a pair of variables, A and B, the causal influence from A to B is represented by the standardized regression coefficients of the path from A at time 1 to B at time 2 (Ccl 1–2). In a similar manner, the causal influence from B to A is represented by the standardized regression coefficients of the path from B at time 1 to A at time 2 (Ccl 2–1). Thus, under the usual assumptions governing regression analysis, a nonzero value of a relevant parameter is indicative of a significant causal effect [48].

All data are expressed and plotted as mean ± SE. P < .05 was considered statistically significant.

## Results

### Creativity scores

**Figure 2:**
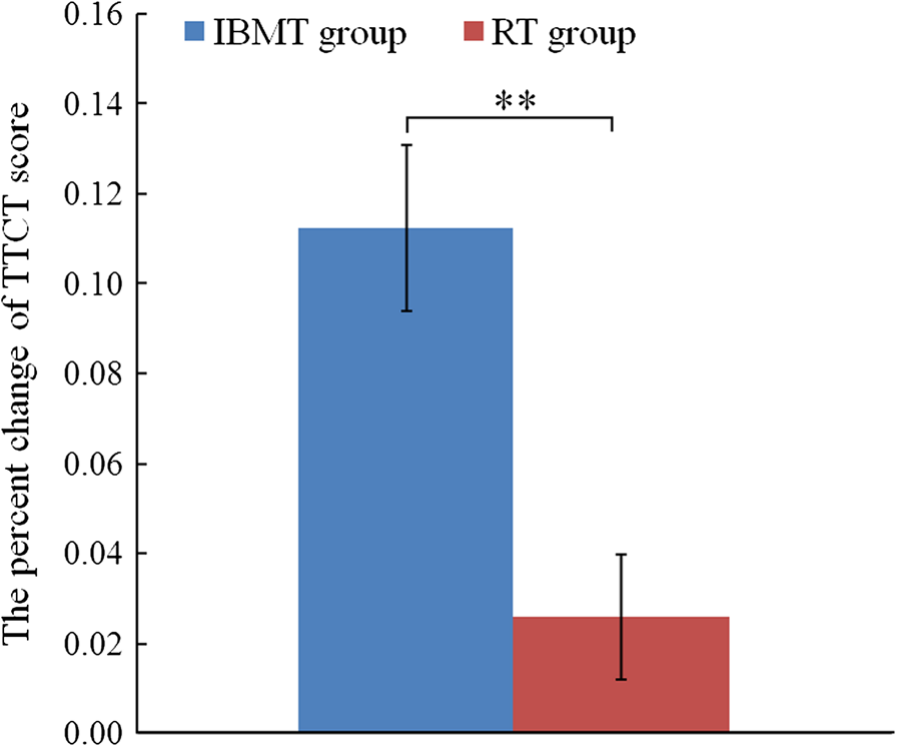
**Comparison of the percent change of TTCT from pre to post between IBMT group and RT group.** IBMT group (blue bars). RT group (red bars). **p < .01. Error bars indicate 1 SE. A higher vertical axis shows a larger improvement of creativity performance.

Before training, the independent t-test showed no significant difference in TTCT (*p* > .05) between the two groups (IBMT:M = 588.950, SE = 7.486; RT:M = 587.32, SE = 11.685). ANOVAs revealed a group (IBMT vs. RT) × session (pre-training vs. post-training) interaction effect [F(1, 37) = 14.853; *p* < .01] and a session (pre-training vs. post-training) main effect [F(1, 37) = 36.156; *p* < .01] for TTCT. The follow-up t-test indicated the IBMT group obtained significantly better scores in TTCT percent change from pre to post (t(37), = 3.755; p < .01) in comparison with the RT group (Figure 2). These results indicated that short-term IBMT can yield a better creative performance than short-term RT.

### Emotion scores

**Figure 3:**
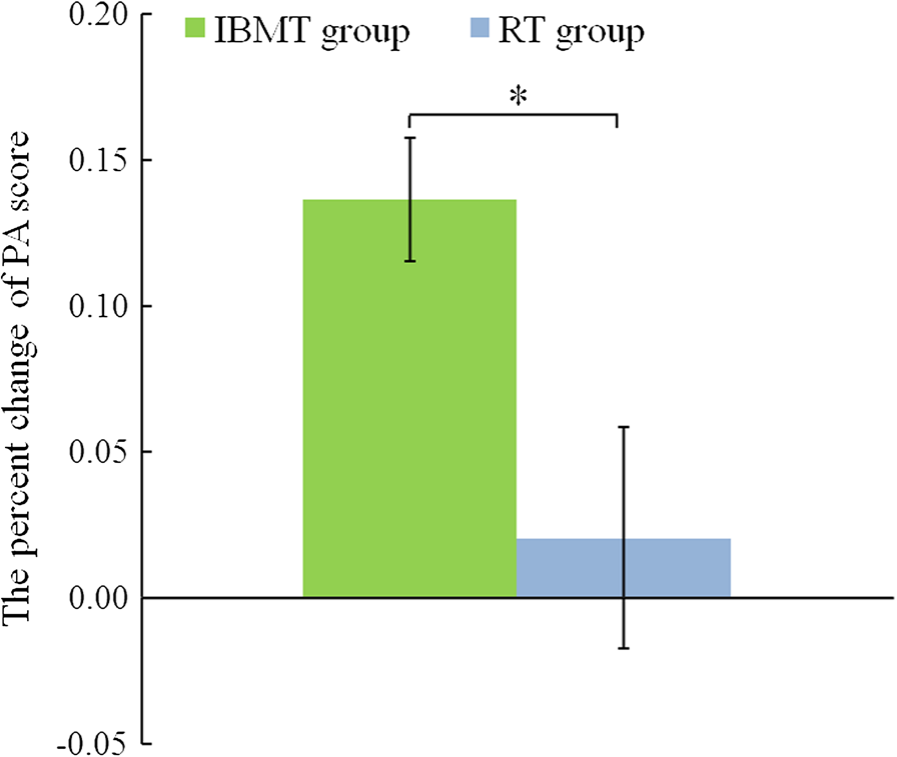
**Comparison of the percent change of PA from pre to post between IBMT group and RT group.** IBMT group (green bar). RT group (blue bar). *p < .05. Error bars indicate 1 SE. A higher vertical axis indicates a larger improvement of mood state.

Before training, the independent t-test showed no significant difference in PA (*p* > .05) between the two groups (IBMT:M = 29.110, SE = .921; RT: M = 29.350, SE = 1.145). The ANOVAs revealed a group (IBMT vs. RT) × session (pre-training vs. post-training) interaction effect [F(1, 37) = 8.941; *p* < .01] and a session (pre-training vs. post-training) main effect [F(1, 37) = 11.603; *p* < .01] for PA. The follow-up t-test indicated the IBMT group obtained significantly better scores in PA percent change from pre to post (t(37) = 2.678; p < .05) in comparison with the RT group (Figure 3). These results suggested that short-term IBMT induces higher positive mood states than RT.

**Figure 4:**
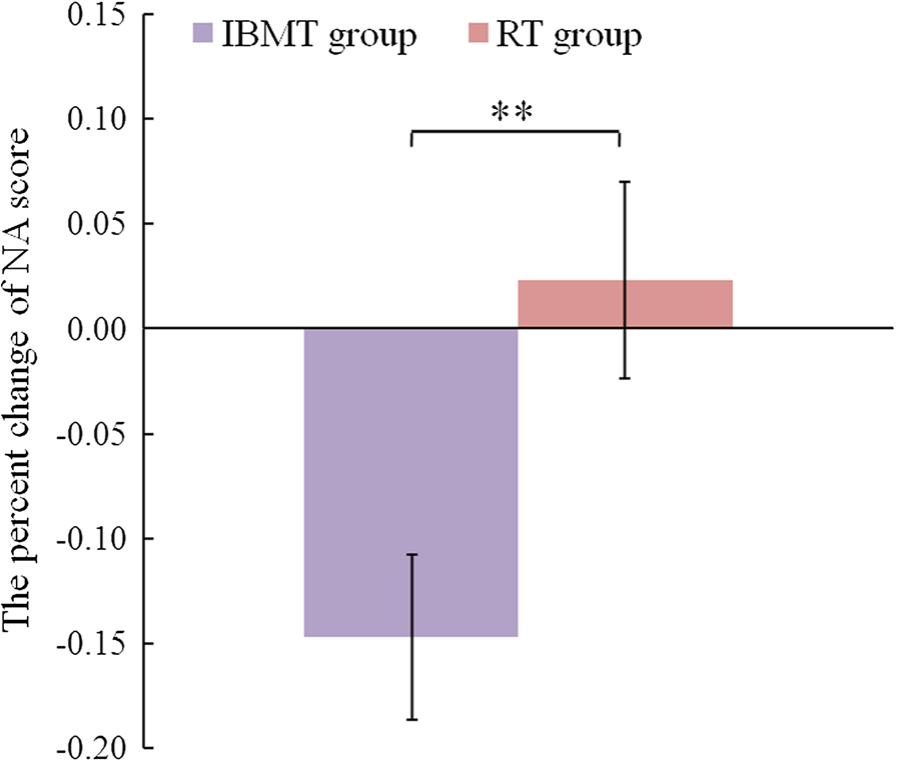
**Comparison of the percent change of PA from pre to post between IBMT group and RT group.** IBMT group (purple bar). RT group (red bar). **p < .01. Error bars indicate 1 SE. A lower vertical axis indicates a larger improvement of mood state.

Before training, the independent t-test showed no significant difference in NA (*p* > .05) between the IBMT group (M = 18.210, SE = .932) and RT group (M = 18.150, SE = .944). ANOVAs revealed a group (IBMT vs. RT) × session (pre-training vs. post-training) interaction effect [F(1, 37) = 8.271; *p* < .01] and a session (pre-training vs. post-training) main effect [F(1, 37) = 8.852; *p* < .01] for NA. The follow-up t-test indicated the IBMT group obtained significantly better scores in NA percent change from pre to post (t(37) = 2.773; *p <* .01) in comparison with the RT group (Figure 4). These results manifested that the short-term IBMT induced lower negative mood states than the short-term RT.

### Relationship between emotion and creativity

To explore the causal sequence between emotion and creativity for short-term training, the PANAS scores and TTCT scores of the IBMT and RT groups across pre-training and post-training sessions were analyzed with cross-lagged panel correlation.

**Figure 5:**
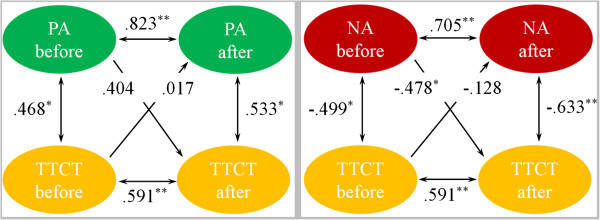
**The Cross-Lagged models for exploring the causal sequence between PANAS and TTCT of IBMT group.** The Cross-Lagged models for exploring the causal sequence between PA score and TTCT score (left panel) and between NA score and TTCT score (right panel) of the IBMT group before and after training. Ellipses indicate measured variables; Arrows depict hypothesized directional or “causal” links/associations; Numbers above or near measured variables represent the correlations. Spearman’s correlation coefficient and the standardized regression coefficient are used and estimates are statistically significant at *p < .05 and **p < .01.

Figure 5 (left panel) shows the Ccls between PA and TTCT in the IBMT group. First, the Csyn coefficients (r PA-before × TTCT-before = .468, r PA-after × TTCT-after = .533) and the Csta coefficients (r PA-before × PA-after = .823, r TTCT-before × TTCT-after = .591) were high in magnitude and statistically significant in the non-cross direction, which provided preliminary support for cross-lagged panel correlation. Second, the standardized regression coefficients of the path from before-training PA score to after-training TTCT score (β = .592; R^2^ = .351; *p* = 0.008) was significant. However, the standardized regression coefficients of the path from after-training PA score to before-training TTCT score (β = .399; R^2^ = .159; *p* = 0.09) was marginally significant. PA had a positive cross-lagged impact on TTCT, and that indicated a causal influence from positive mood changes to the creativity changes in IBMT group. In addition, Figure 5 (right panel) shows the Ccls between NA and TTCT in the IBMT group. First, the Csyn coefficients (r NA-before × TTCT-before = −.499, r NA-after × TTCT-after = −.633) and the Csta coefficients (r NA-before × NA-after = .705, r TTCT-before × TTCT-after = .591) were high in magnitude and statistically significant in the non-cross direction, which provided preliminary support for cross-lagged panel correlation. Second, the standardized regression coefficients of the path from before-training NA score to after-training TTCT score (β = −.654; R^2^ = .427; p = 0.002) was significant. However, the standardized regression coefficients of the path from after-training NA score to before-training TTCT score (β = −.256; R^2^ = .065; p = 0.291) was not significant. NA had a negative cross-lagged impact on TTCT, and that indicated a causal influence from negative mood changes to the creativity changes in the IBMT group.

**Figure 6:**
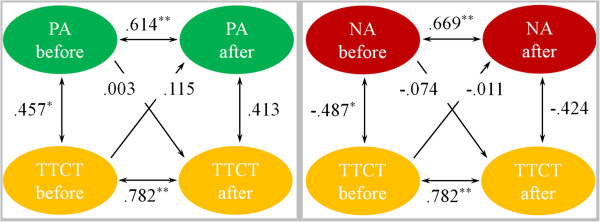
**The Cross-Lagged models for exploring the causal sequence between PANAS and TTCT of RT group.** The Cross-Lagged models for exploring the causal sequence between PA score and TTCT score (left panel) and between NA score and TTCT score (right panel) of the RT group before and after training.

Figure 6 (left panel) shows the Ccls between PA and TTCT in the RT group. As a preliminary analysis step, Csyn coefficient (r PA-before × TTCT-before = .457) presented to be significant, whereas Csyn coefficient (r PA-after × TTCT-after = .413) was not significant, suggesting that Csyn coefficient did not support cross-lagged panel correlation. Thus, positive mood changes were not the cause of the creativity changes found in the short-term RT group. In addition, Figure 6 (right panel) shows the Ccls between NA and TTCT in RT group. As a preliminary analysis step, Csyn coefficient (r NA-before × TTCT-before = −.487) presented to be significant, whereas Csyn coefficient (r NA-after × TTCT-after = −.424) was not significant, suggesting that Csyn coefficient did not support cross-lagged panel correlation. Thus, negative mood changes were not the cause of the creativity changes found in the short-term RT group.

To summarize, the cross-lagged analyses indicated that both positive and negative mood changes may contribute to the creativity changes in the short-term IBMT group, but not in the RT group.

## Discussion

Consistent with our previous research [19], the IBMT group significantly outperformed the RT group in TTCT scores after training. TTCT is used to evaluate creativity through divergent thinking [55], which is a key aspect of creativity and predictor of creative ability [56]. We concluded that creative performance on the divergent thinking task was better following IBMT than RT. Thus, the results are consistent with our hypotheses. The improvement of creativity may be caused by a variety of factors. Previous studies have shown that there may be a wealth of psychological factors, such as intelligence, self-confidence, attention, cognitive flexibility [57] and mood states [21,22,25,26] with regard to the influence on creative fluency and originality.

In the PANAS, the PA score (assessed by PA subscale) increased significantly and the NA score (by NA subscale) decreased significantly after 3.5 hours of IBMT compared to RT. We concluded that short-term IBMT yielded a better emotion state than RT. Thus, the results are consistent with our hypotheses that the IBMT group improved emotional regulation, whereas RT group did not.

Moreover, we hypothesized that emotional improvement may be one way that TTCT scores are changed in short-term meditation. Our results revealed that the cross-lagged analysis documented PA and NA as an antecedent of creativity in the IBMT group. The standardized regression coefficients of the path from before-training PA (or NA) to after-training TTCT was significant, while the standardized regression coefficients of the path from after-training PA (or NA) to before-training TTCT was marginally significant. However, similar effects of emotion on creativity were not found in the RT group. Our results indicated that emotion-based creativity-promoting mechanism is attributed to IBMT.

Creativity includes a wide range of cognitive processes, such as flow (when a person is fully immersed in what s/he is doing, characterized by a feeling of energized focus, full involvement, and success in the process of the activity) [58], breadth of attention [59], and remote association of ideas [1]. However, emotion is associated with these cognitive processes that contribute to the complex of creativity [21,22,25,26]. One mood theory is that PA promotes a more global scope of attention [60,61], enhancing access to distant or unusual associations [62,63], which facilitates creative solutions to classic creative problems such as improving performance [64] on the Remote Associates Test [65]. Another mood theory is that PA enhances switching between global and local attention modes [66] or between strategies [67], or in other words that it enhances selection of different perspectives [27]. In contrast, NA states such as anxiety and depression are associated with deficits in attentional and cognitive control mechanisms [68,69], often inducing a narrow scope of attention [70]. Therefore, NA states should impede cognitive flexibility and creative problem solving.

## Conclusions

Taken together, these results support our hypothesis that creative performance on the divergent thinking task and emotion were better following IBMT than RT, and meditation with mood regulation effects have potential benefit to levels of creativity. Our study may open up an important avenue for research into the relationships between meditation - emotion - creativity.

## Abbreviations

PANAS: Positive and negative affect schedule; TTCT: Torrance tests of creative thinking; IBMT: Integrative body-mind training; RT: Relaxation training; PA: Positive affect; NA: Negative affect; DUT: Dalian University of technology; Csta: Stationarity of correlations; Csyn: Synchronous correlations; Ccl: Cross-lagged correlations; ANOVA: Analysis of variance; SE: Standard error mean.

## Competing interests

The authors declare that they have no competing interests.

## Authors’ contributions

YYT and MIP designed and supervised the study. XQD contributed to data acquisition, analysis and interpretation. YYT, MIP, RXT and XQD drafted and revised the manuscript. All authors read and approved the final manuscript.
